# Clinical cross-sectional diagnostic accuracy study of DIAGNOdent Pen and QrayCam Pro quantitative fluorescence for occlusal caries detection and ICDAS II-derived lesion categorization in permanent posterior teeth

**DOI:** 10.1186/s12903-026-09180-y

**Published:** 2026-07-04

**Authors:** Ahmad Bittar, Elif Alkan, Dilek Tağtekin

**Affiliations:** 1 Department of Restorative Dentistry, Faculty of Dentistry, İstinye University, Istanbul, Türkiye; 2 Department of Restorative Dentistry, Faculty of Dentistry, Marmara University, Istanbul, Türkiye

**Keywords:** Occlusal caries, DIAGNOdent Pen, QrayCam Pro, Quantitative light-induced fluorescence, ICDAS II, Lesion categorization

## Abstract

**Background:**

Early detection of occlusal caries in posterior teeth is important for minimally invasive care. Visual systems such as the International Caries Detection and Assessment System (ICDAS II) standardize clinical scoring, but adjunctive fluorescence-based tools may improve objectivity. This clinical cross-sectional diagnostic accuracy study evaluated DIAGNOdent Pen and QrayCam Pro metrics (ΔFmax and ΔRmax) relative to ICDAS II-derived lesion categories in permanent posterior teeth.

**Methods:**

This clinical cross-sectional diagnostic accuracy study included 64 adults who contributed 145 unrestored posterior teeth; the tooth was the analytical unit because all measurements were site-specific. After prophylaxis and air-drying, calibrated examiners assigned ICDAS II scores, obtained DIAGNOdent Pen peak readings, and captured QrayCam Pro images at the same sites. QrayCam Pro analysis yielded maximum fluorescence loss (ΔFmax) and maximum red fluorescence gain (ΔRmax, %). ICDAS II scores were grouped as E0 (0), E1 (1), E2 (2–3), and D1 (4–5). Spearman correlation and ROC analyses were performed.

**Results:**

Thirty sound surfaces and 115 ICDAS II-detected lesions were analysed. Median DIAGNOdent values increased from 10.5 in E0 to 74.0 in D1, ΔFmax decreased from 0.0% to − 60.0%, and ΔRmax increased from 0.0% to 70.0%. ICDAS II correlated strongly with DIAGNOdent (ρ = 0.91) and ΔFmax (ρ = −0.80) and moderately with ΔRmax (ρ = 0.67) (all p < 0.001). For E0 vs. (E1 + E2+D1), AUCs were 0.94 for DIAGNOdent, 0.97 for ΔFmax, and 0.74 for ΔRmax. For E2 vs. D1, AUCs were 0.93, 0.85, and 0.80, respectively.

**Conclusions:**

In this exploratory tooth-level analysis, DIAGNOdent Pen and QrayCam Pro metrics showed descriptive agreement with ICDAS II-derived clinical categories. Because analyses were not adjusted for within-participant clustering and formal pairwise AUC comparisons were not performed, the observed AUC patterns should be interpreted cautiously. The devices appear to provide complementary adjunctive information, and proposed cut-offs require external validation before clinical use.

**Supplementary Information:**

The online version contains supplementary material available at 10.1186/s12903-026-09180-y.

## Background

Occlusal caries in posterior teeth remain a leading reason for restorative treatment, despite progress in preventive dentistry and fluoride exposure. Accurate detection and clinical categorization of these lesions are essential for preserving tooth structure and supporting non-invasive or micro-invasive strategies instead of early operative interventions [1].

The International Caries Detection and Assessment System (ICDAS II) was developed to standardize visual scoring and provide an ordinal scale for early and advanced caries compared with traditional sound/decayed criteria [2]. In vitro and clinical studies have shown that ICDAS II has good reproducibility, diagnostic validity, and correlation with histological depth, but detection of early lesions and recognition of signs suggestive of dentin involvement remain examiner-dependent and require calibration [3–5]. In daily practice, visual-tactile examination alone may therefore under-detect subtle lesions or underestimate lesion severity.

Laser fluorescence devices, particularly the DIAGNOdent Pen (KaVo; Biberach, Germany), were introduced to provide an objective numeric adjunct for occlusal caries detection by exploiting fluorescence from bacterial metabolites and altered tooth structure [6–8]. Laboratory and clinical studies have reported good reproducibility and moderate-to-high accuracy of DIAGNOdent for occlusal caries detection, although performance varies across studies and lesion thresholds and the device does not directly assess lesion activity [6–11].

Quantitative light-induced fluorescence (QLF) systems use blue-light excitation and digital image analysis to quantify changes in enamel fluorescence, typically expressed as fluorescence loss (ΔF) relative to sound reference tissue [12–13]. More recently, QLF cameras such as QrayCam Pro have incorporated red fluorescence measurements (ΔR), expressed as the percentage increase in the red/green fluorescence ratio relative to sound tissue and associated with porphyrin-related fluorescence from mature plaque/biofilm; however, ΔR should not be interpreted as a direct surrogate for histological lesion extension [14–15]. QLF has been used clinically to monitor early enamel lesions and responses to non-invasive care, but less often for chairside lesion categorization [12–13].

Several studies have compared visual scoring, DIAGNOdent and QLF for occlusal or proximal caries detection, mostly in vitro or in primary teeth [10–11, 16]. However, clinical evidence comparing DIAGNOdent Pen with a modern QLF camera such as QrayCam Pro—using both ΔF and ΔR metrics—for occlusal caries detection in permanent posterior teeth remains limited. In addition, most available data focus on simple carious-versus-sound outcomes rather than ICDAS II-derived severity groupings that may influence preventive, micro-invasive, or restorative management.

An in vitro study by Savas et al. compared ICDAS II, DIAGNOdent, Midwest Caries ID and QLF for occlusal caries in primary molars and reported AUC values between 0.82 and 0.88 for DIAGNOdent and 0.86 to 0.88 for QLF at enamel and dentin thresholds [17]. That study used histology as the reference standard, relied on extracted teeth, and did not include red-fluorescence metrics.

Against this background, the aim of this clinical cross-sectional diagnostic accuracy study was to compare the strength of association and discriminatory performance of DIAGNOdent Pen and QrayCam Pro quantitative fluorescence metrics (ΔFmax and ΔRmax), each interpreted against ICDAS II-derived lesion categories, in permanent posterior teeth. The predefined clinical contrasts were sound versus any ICDAS II-detected lesion, initial enamel changes versus the remaining categories, and enamel-category versus dentin-suspected lesions.

## Methods

### Study design and setting

This single-centre clinical cross-sectional diagnostic accuracy study was conducted in accordance with the Declaration of Helsinki and was approved by the Marmara University Faculty of Dentistry Ethics Committee for Clinic-Related Studies (decision date 03 July 2025, approval no. 2025-08-02/2025-46). Written informed consent was obtained from all participants. The study was not prospectively registered because it was observational and non-interventional.

### Participants and tooth selection

Adult patients attending the clinic for routine care or caries management were screened consecutively for eligibility. The occlusal surface/tooth was prespecified as the analytical unit because ICDAS II, DIAGNOdent Pen and QrayCam Pro outputs were all site-specific. More than one tooth could therefore be included per participant. 

Inclusion criteria were:

- Age ≥ 18 years.
- Presence of at least one permanent posterior tooth (premolar or molar) with an unrestored occlusal surface.
- Occlusal ICDAS II code 0–5 (sound to advanced non-cavitated/cavitated lesions confined to the occlusal surface).

Exclusion criteria were:

- Teeth with occlusal restorations, fissure sealants, hypoplastic defects or developmental enamel anomalies.
- Teeth with gross fractures, extensive wear or structural defects precluding reliable fluorescence readings.
- Teeth with ICDAS II code 6 lesions (extensive cavitation into dentin).
- Patients with fixed orthodontic appliances on the posterior teeth of interest.

**Fig. 1:**
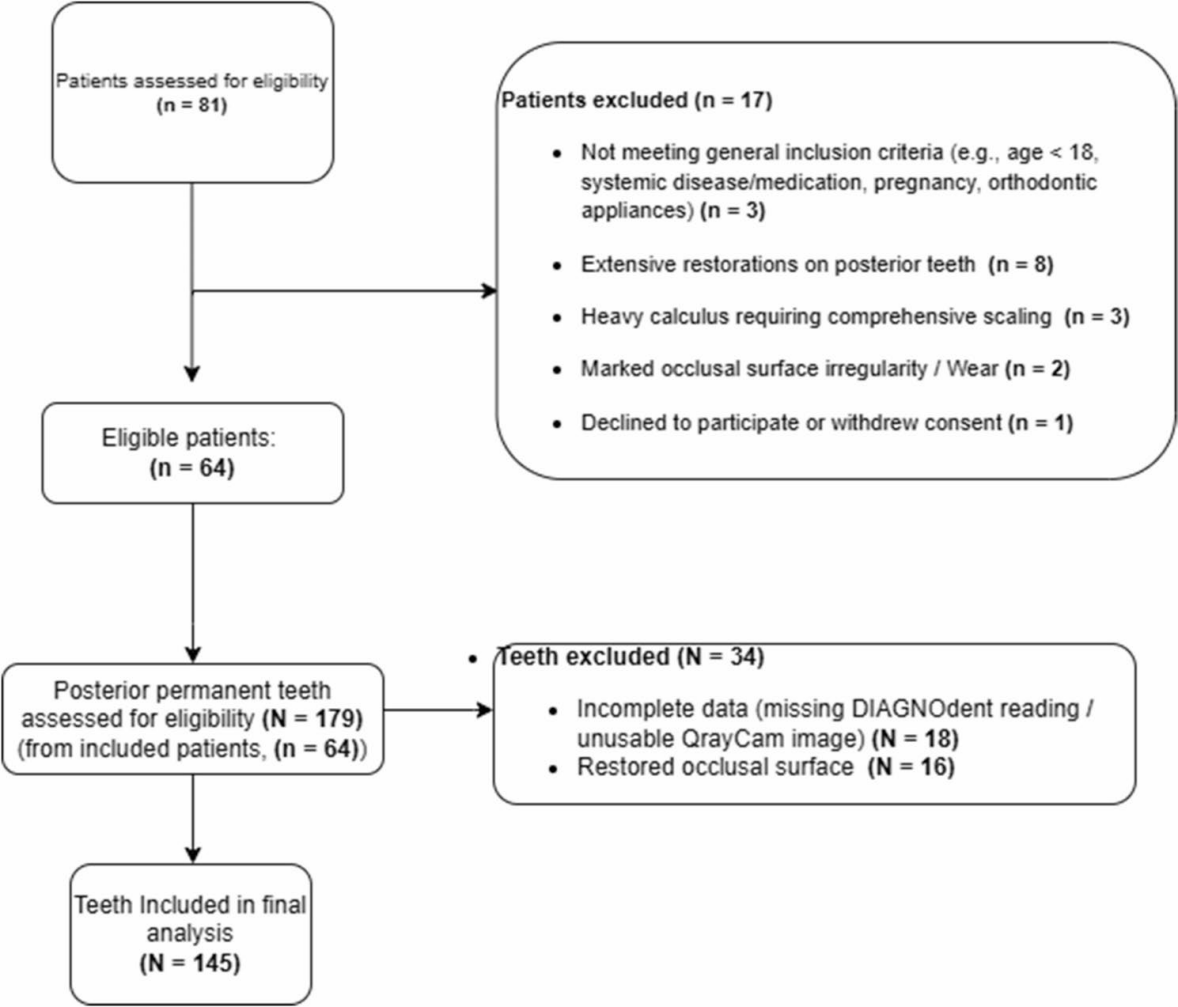
Flowchart of patient and tooth inclusion. Flow diagram showing the number of patients screened, excluded (with reasons) and included, and the distribution of included teeth across ICDAS II occlusal codes 0–5 (total 64 patients and 145 posterior teeth) n = patients; N = teeth

A total of 64 patients (mean age 26.3 years [SD 5.2]; 36 females, 28 males) were included, contributing 145 posterior permanent teeth (one to four teeth per patient). The distribution across ICDAS II categories was not quota-controlled and reflects the clinical case mix encountered during recruitment. Patient enrolment and tooth selection are summarised in Fig. 1.

No formal a priori sample-size calculation tailored to ROC endpoints was performed because reliable pilot estimates for expected AUCs and within-participant clustering were unavailable during study planning. The study therefore should be regarded as exploratory, and post hoc power was not calculated because effect estimates with 95% confidence intervals are more informative than retrospective power calculations [18].

### Examiner calibration and ICDAS II scoring

Two examiners were trained and calibrated in ICDAS II scoring using standard online resources and reference images [2]. Calibration was performed on a separate set of 30 occlusal surfaces not included in the final sample. Each examiner scored the training set twice, two weeks apart.

Inter- and intra-examiner agreement for ICDAS II scores in the calibration set were 0.96 and 0.88, respectively, indicating excellent consistency. After calibration, all study teeth were examined clinically under overhead dental light following professional prophylaxis and drying with oil-free air for 5 s. The highest ICDAS II code observed on the occlusal surface was recorded for each tooth.

For tooth-level group comparisons, correlation analyses, and ROC analyses, ICDAS II codes were also grouped into four ICDAS II-derived lesion categories as a clinical severity surrogate:

- E0: sound (ICDAS II code 0).
- E1: initial enamel changes (ICDAS II code 1).
- E2: established enamel category (ICDAS II codes 2–3).
- D1: dentin-suspected category (ICDAS II codes 4–5).

### DIAGNOdent pen measurements

After ICDAS II scoring, laser fluorescence measurements were obtained using the DIAGNOdent Pen (KaVo; Biberach, Germany) according to the manufacturer’s instructions. The device was calibrated with its ceramic reference block before each patient. For each tooth, the tip was positioned on the central fissure and gently moved over the occlusal surface without pressure to identify the site with the highest reading.

**Fig. 2:**
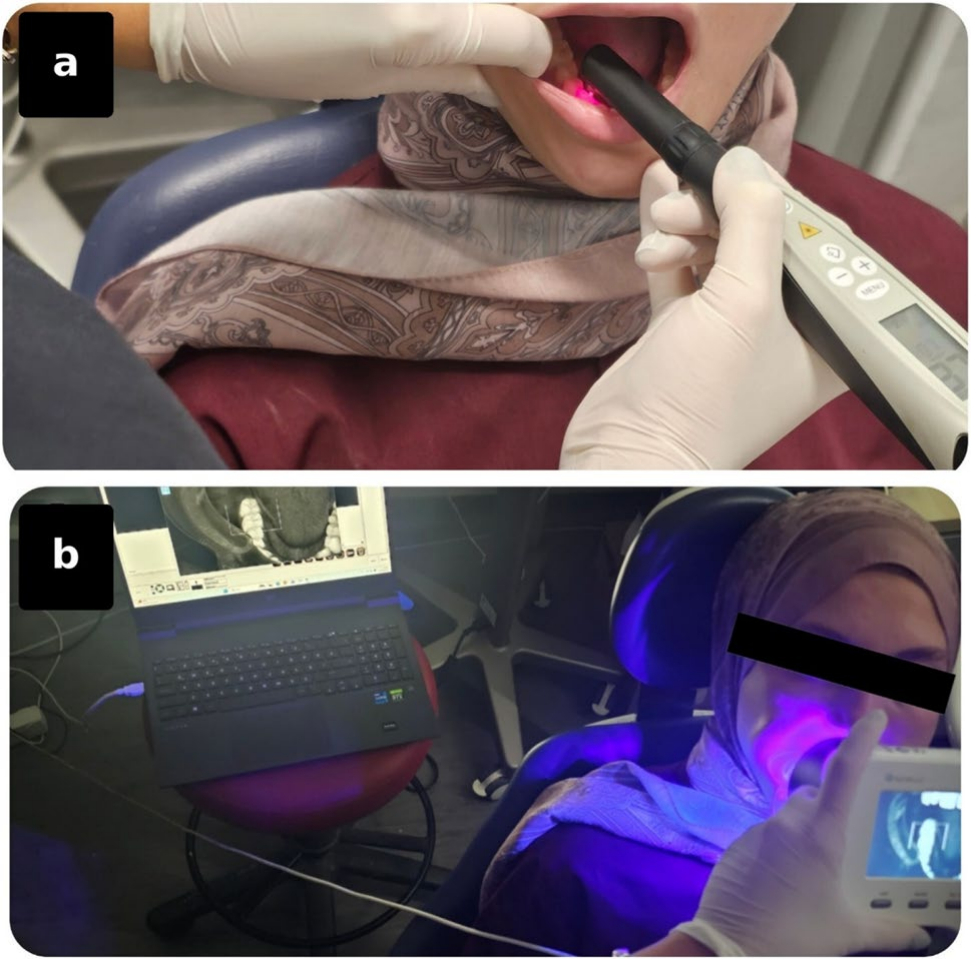
Clinical use of DIAGNOdent Pen and QrayCam Pro. **a** DIAGNOdent Pen measurement on an occlusal surface after professional prophylaxis and gentle air-drying. **b** Acquisition of occlusal fluorescence images with the QrayCam Pro in a darkened operatory (overhead dental light switched off and ambient operatory lights dimmed); soft tissues are retracted while the camera is positioned perpendicular to the occlusal surface to optimise ΔFmax and ΔRmax measurements

Three consecutive scans were obtained at the same site. During each DIAGNOdent Pen scan, the clinician moved/rotated the tip and recorded the peak value. The mean of the three peak readings was used for analysis [6–8]. Care was taken to minimise plaque, stains and calculus by performing prophylaxis and rinsing before measurement. DIAGNOdent measurements were performed after ICDAS II scoring and the examiner was not blinded to ICDAS II codes; this is addressed as a limitation (Fig. 2).

### QrayCam pro imaging and image analysis

Following DIAGNOdent measurements, occlusal images were acquired using a QrayCam Pro device (AIOBIO; Seoul, Republic of Korea). The camera was positioned perpendicular to the occlusal surface at a standardised distance with the manufacturer’s positioning guide. To minimise ambient-light interference, the overhead dental light was switched off and imaging was performed in a darkened operatory (Fig. 2).

**Fig. 3:**
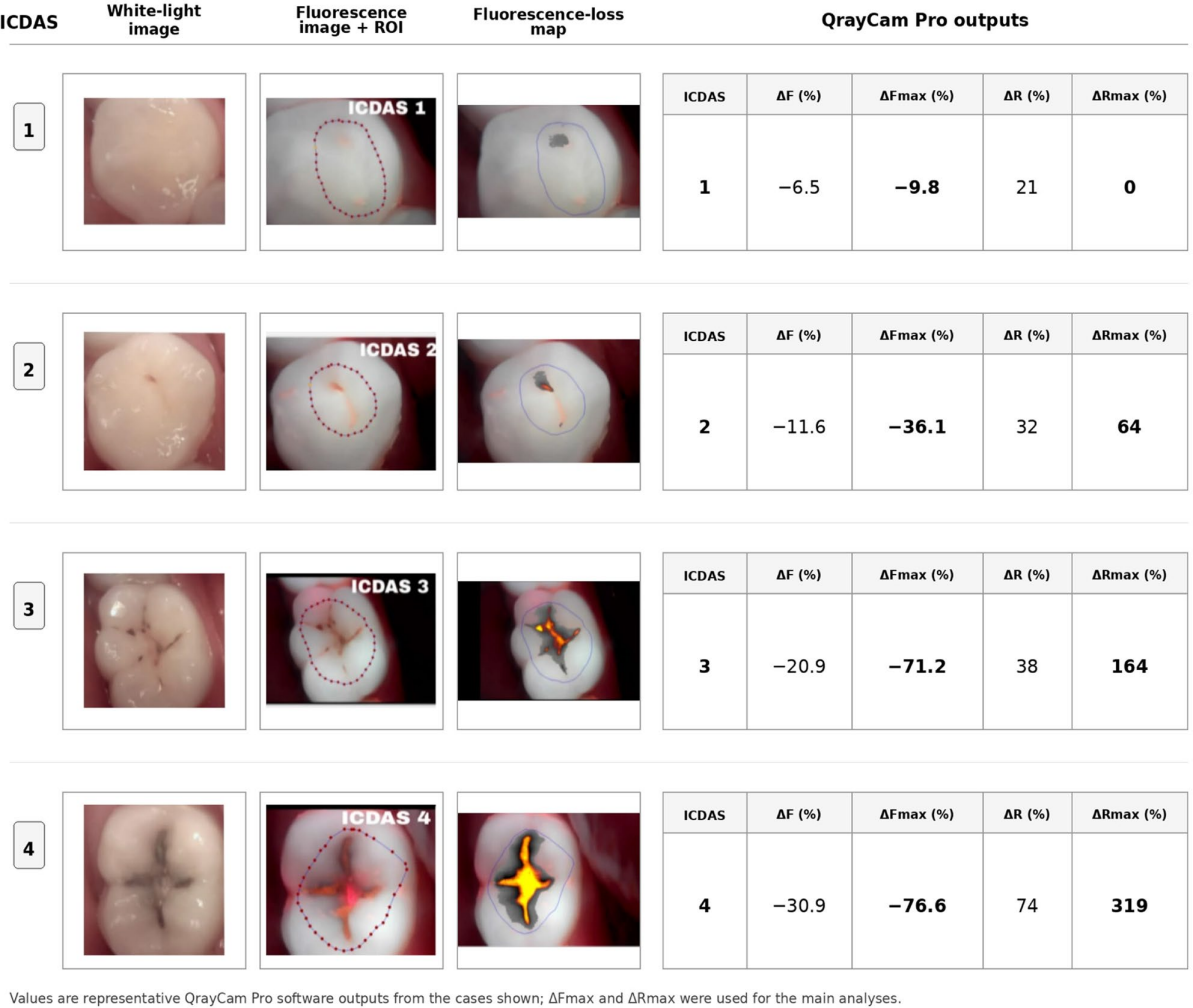
QrayCam Pro quantitative fluorescence workflow across ICDAS categories. Representative cases are arranged sequentially from ICDAS 1 to ICDAS 4. From left to right, each row shows the white-light image, fluorescence image with the manually defined ROI, the software-identified fluorescence-loss map within the ROI, and a standardized table of the corresponding QrayCam Pro outputs. The numerical values displayed in the right-hand table are also listed in Supplementary Table S3 to improve readability. ΔFmax and ΔRmax were recorded as the maximum values within the ROI

For each tooth, a white-light and corresponding fluorescence image were captured. All images were exported and analysed using the manufacturer’s software (Q-ray Clinical, version 1.45; AIOBIO; Seoul, Republic of Korea). Regions of interest (ROIs) were placed according to a predefined protocol: (1) identify the clinically most suspicious pit/fissure area on the occlusal surface; (2) draw a closed ROI that encloses the main pit/fissure system while avoiding adjacent teeth and specular highlights; and (3) trace the ROI boundary predominantly on surrounding visually sound enamel, rather than along fissure grooves or areas of suspected mineral loss, to provide a local sound-enamel reference for fluorescence comparison. An illustrative example of the analysis workflow is provided in Fig. 3. The maximum values within the ROI were recorded as ΔFmax and ΔRmax.

The following parameters were extracted:

- ΔFmax (%): maximum percentage fluorescence loss within the region of interest relative to surrounding sound enamel (negative values indicate loss).
- ΔRmax (%): maximum percentage increase in the red/green fluorescence ratio within the region of interest relative to surrounding sound enamel.

Each tooth therefore contributed one DIAGNOdent value, one ΔFmax and one ΔRmax.

### Outcome definition

Because histological validation is not feasible in a clinical cross-sectional study, ICDAS II-derived lesion categories (E0, E1, E2, D1) were used as the clinical comparator for diagnostic performance analyses. These categories were not treated as direct histological depth categories. Three diagnostic thresholds were defined:

1. Any lesion vs. sound: E0 vs. (E1 + E2+D1)
2. Initial enamel vs. other stages: E1 vs. (E0 + E2+D1)
3. Enamel-category vs. dentin-suspected category: E2 vs. D1.

These thresholds were chosen to reflect clinically relevant contrasts (sound vs. any ICDAS II-detected lesion; initial enamel changes vs. remaining categories; and enamel-category vs. dentin-suspected category). Device outputs were interpreted as adjunctive information rather than standalone treatment-decision thresholds.

### Statistical analysis

Statistical analyses were performed in IBM SPSS Statistics for Windows, version 25.0 (IBM; Armonk, NY, USA). Continuous variables were summarised as median (min–max) because DIAGNOdent, ΔFmax and ΔRmax were non-normally distributed. Categorical variables were summarised as counts and percentages. Differences in device metrics across ICDAS II codes and ICDAS II-derived lesion categories were assessed with Kruskal–Wallis tests with post-hoc pairwise comparisons where appropriate. Associations between ICDAS II scores, ICDAS II-derived categories, DIAGNOdent, ΔFmax and ΔRmax were assessed using Spearman rank correlation coefficients (ρ) with two-sided p-values. ROC analyses were performed for the three predefined diagnostic thresholds; AUCs and 95% confidence intervals were estimated non-parametrically, and exploratory cut-offs for the primary threshold were derived from the maximum Youden index. Analyses were performed at the tooth level without cluster adjustment for multiple teeth from the same participant; therefore, confidence intervals and p-values should be interpreted as exploratory.

## Results

### Patient and tooth characteristics

Sixty-four patients (mean age 26.3 years [SD 5.2]; 36 females, 28 males) contributed 145 posterior teeth with ICDAS II codes 0–5. The tooth was the analytical unit. The ICDAS II distribution was:

- 30 sound occlusal surfaces (code 0; 20.7%).
- 39 code 1 lesions (26.9%).
- 12 code 2 lesions (8.3%).
- 36 code 3 lesions (24.8%).
- 23 code 4 lesions (15.9%).
- 5 code 5 lesions (3.4%).

No teeth had ICDAS II code 6. When grouped into ICDAS II-derived lesion categories, there were 30 E0 (20.7%), 39 E1 (26.9%), 48 E2 (33.1%) and 28 D1 (19.3%) surfaces (Table 1).

**Table 1:** DIAGNOdent pen values and QrayCam Pro ΔFmax and ΔRmax across ICDAS II-derived lesion categories (E0–D1)

Group	Definition (ICDAS II)	n	DIAGNOdent Pen, median (min–max)	ΔFmax (%), median (min–max)	ΔRmax (%), median (min–max)
E0	Sound (code 0)	30	10.5 (2–16)	0.0 (−27 to 0)	0.0 (0–0)
E1	Initial enamel (code 1)	39	14.0 (6–25)	−17.0 (−53 to 0)	0.0 (0–48)
E2	Established enamel (2–3)	48	40.0 (18–99)	−37.5 (−65 to −9)	38.0 (0–246)
D1	Dentin-suspected (4–5)	28	74.0 (30–99)	−60.0 (−78 to −9)	70.0 (0–297)
Total		145	21.0 (2–99)	−27.0 (−78 to 0)	0.0 (0–297)

### Fluorescence metrics by ICDAS II code and ICDAS II-derived lesion categories

DIAGNOdent, ΔFmax and ΔRmax all showed monotonic changes across ICDAS II codes (Supplementary Table S1). Median DIAGNOdent values increased from 10.5 (2–16) for sound occlusal surfaces (ICDAS II code 0) to 14.0 (6–25) for code 1, 23.0 (20–40) for code 2, 45.5 (18–99) for code 3, 68.0 (30–99) for code 4 and 99.0 (67–99) for code 5 lesions.

ΔFmax became progressively more negative with increasing ICDAS II score, from 0.0% (− 27 to 0) in sound teeth to − 17.0% (− 53 to 0) in code 1, − 34.0% (− 65 to − 14) in code 2, − 38.5% (− 65 to − 9) in code 3, − 60.0% (− 77 to − 9) in code 4 and − 65.0% (− 78 to − 13) in code 5. Thus, greater fluorescence loss (more negative ΔFmax) corresponded to visually more advanced lesions.

ΔRmax remained close to zero in sound and early enamel lesions (median 0.0% in ICDAS II 0–2) but increased in more advanced clinical categories, reaching 41.5% (0–246%) in code 3, 68.0% (0–149%) in code 4 and 281.0% (0–297%) in code 5 (Supplementary Table S1). This pattern is consistent with ΔR capturing biofilm-associated red fluorescence that tends to be more pronounced in clinically advanced or plaque-retentive sites, rather than directly representing histological lesion extension.

A similar gradient was observed when teeth were grouped by ICDAS II-derived lesion categories (E0–D1) (Table 1). Median DIAGNOdent increased from 10.5 in E0 to 14.0 in E1, 40.0 in E2 and 74.0 in D1, while median ΔFmax decreased from 0.0% to − 17.0%, − 37.5% and − 60.0%, respectively. ΔRmax rose from 0.0% (E0 and E1) to 38.0% (E2) and 70.0% (D1).

### Correlations between visual and fluorescence-based measures

Spearman correlation coefficients are summarised in Table 2. DIAGNOdent showed very strong positive correlations with ICDAS II (ρ = 0.91, *p* < 0.001) and ICDAS II-derived category (E0–D1; ρ = 0.90, *p* < 0.001). ΔFmax exhibited comparably strong but inverse correlations with ICDAS II (ρ = −0.80, *p* < 0.001) and ICDAS II-derived category (ρ = −0.80, *p* < 0.001), reflecting increasing fluorescence loss with higher ICDAS II scores.

**Table 2:** Spearman correlation coefficients (ρ) between clinical and fluorescence-based measures

Pair of variables	Spearman’s ρ	*p*-value
DIAGNOdent vs ΔFmax	−0.779	<0.001
DIAGNOdent vs ΔRmax	0.671	<0.001
DIAGNOdent vs ICDAS II-derived category (E0–D1)	0.895	<0.001
DIAGNOdent vs ICDAS II	0.912	<0.001
ΔFmax vs ΔRmax	−0.755	<0.001
ΔFmax vs ICDAS II-derived category	−0.802	<0.001
ΔFmax vs ICDAS II	−0.797	<0.001
ΔRmax vs ICDAS II-derived category	0.655	<0.001
ΔRmax vs ICDAS II	0.666	<0.001
ICDAS II-derived category vs ICDAS II	0.988	<0.001

ICDAS II-derived category (E0–D1) = four-level ordinal scale derived from ICDAS II codes (E0: 0; E1: 1; E2: 2–3; D1: 4–5)

ΔRmax correlated moderately-to-strongly with ICDAS II (ρ = 0.67, *p* < 0.001) and ICDAS II-derived category (ρ = 0.66, *p* < 0.001), but the coefficients were consistently lower than those for DIAGNOdent and ΔFmax. This suggests that red fluorescence provides complementary information that is more strongly influenced by surface biofilm characteristics and lesion severity than by the ICDAS category alone.

As expected, DIAGNOdent and ΔFmax were strongly inversely correlated (ρ = −0.78, *p* < 0.001), while ΔFmax and ΔRmax showed a strong negative relationship (ρ = −0.76, *p* < 0.001). These patterns indicate that all three metrics are capturing overlapping but not identical aspects of clinical lesion severity and surface biofilm-associated fluorescence.

### ROC analysis

**Fig. 4:**
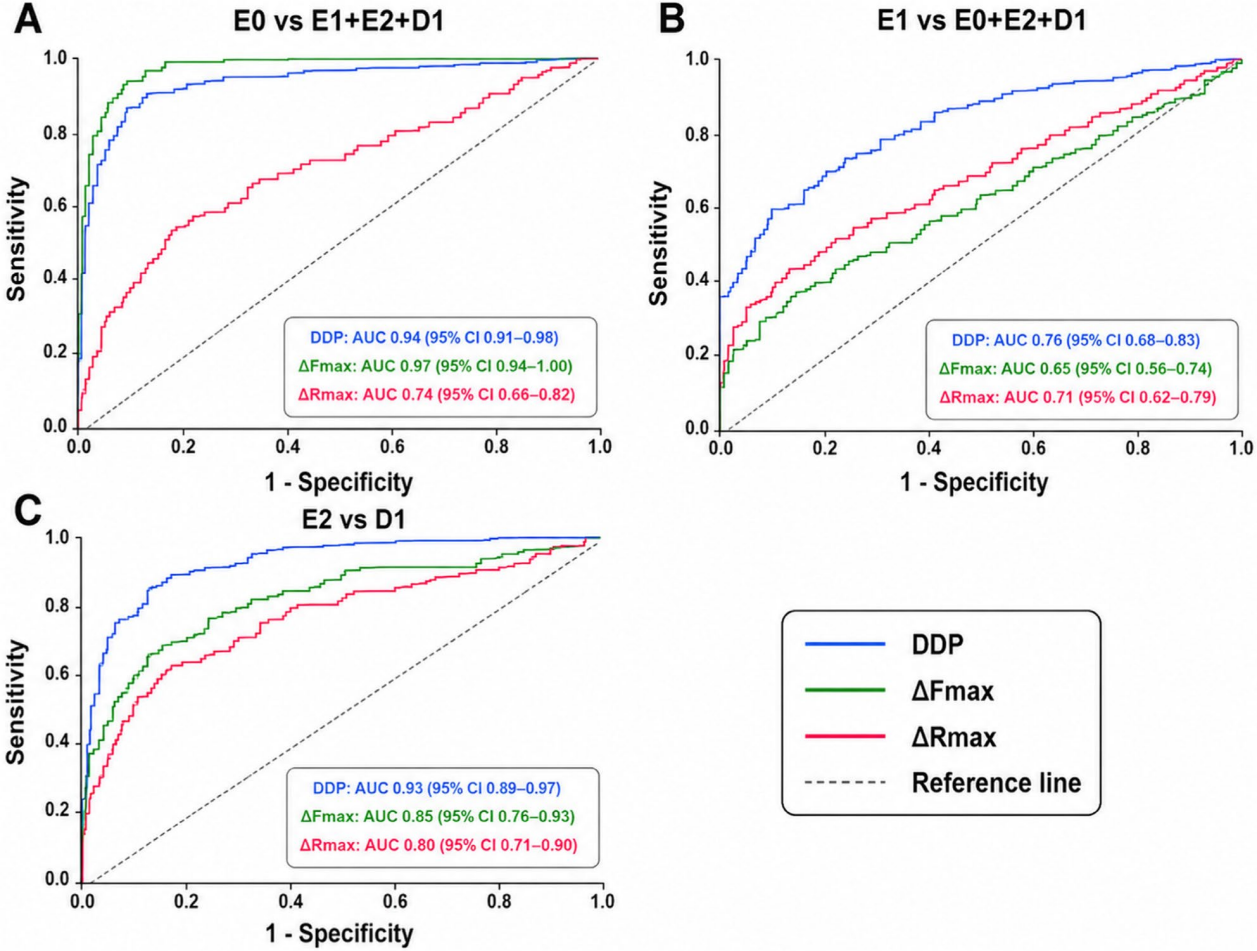
Multi-panel ROC curves for the predefined diagnostic thresholds. Panel **A** shows E0 vs. (E1 + E2+D1), panel **B** shows E1 vs. (E0 + E2+D1), and panel **C** shows E2 vs. D1. Curves are shown for DIAGNOdent Pen (DDP), ΔFmax, and ΔRmax. AUC values with 95% confidence intervals are annotated within each panel. Curves were plotted using empirical ROC coordinates (sensitivity vs. 1 − specificity) from the non-parametric ROC analysis

AUC values for discrimination at the three diagnostic thresholds are shown in Table 3, and the corresponding ROC curves are presented as a multi-panel composite in Fig. 4. For the E0 vs. (E1 + E2+D1) threshold, AUCs were 0.97 (95% CI 0.94–1.00) for ΔFmax, 0.94 (0.91–0.98) for DIAGNOdent and 0.74 (0.66–0.82) for ΔRmax. For the E1-versus-others threshold, AUCs were 0.76 (0.68–0.83) for DIAGNOdent, 0.65 (0.56–0.74) for ΔFmax and 0.71 (0.62–0.79) for ΔRmax. For E2 vs. D1, AUCs were 0.93 (0.89–0.97), 0.85 (0.76–0.93) and 0.80 (0.71–0.90), respectively.

**Table 3:** Area under the ROC curve (AUC) and 95% confidence intervals for DIAGNOdent Pen and QrayCam Pro metrics at different diagnostic thresholds

Threshold (outcome)	Metric	AUC (95% CI)
E0 vs. (E1 + E2+D1)	DIAGNOdent	0.94 (0.91–0.98)
	ΔFmax	0.97 (0.94–1.00)
	ΔRmax	0.74 (0.66–0.82)
E1 vs. (E0 + E2+D1)	DIAGNOdent	0.76 (0.68–0.83)
	ΔFmax	0.65 (0.56–0.74)
	ΔRmax	0.71 (0.62–0.79)
E2 vs. D1	DIAGNOdent	0.93 (0.89–0.97)
	ΔFmax	0.85 (0.76–0.93)
	ΔRmax	0.80 (0.71–0.90)

These ROC findings are descriptive. In this exploratory sample, the numerically largest AUCs were observed for ΔFmax at the sound-versus-lesion threshold and for DIAGNOdent at the E2-versus-D1 threshold; however, formal pairwise AUC comparison testing was not performed.

## Discussion

This clinical cross-sectional diagnostic accuracy study compared DIAGNOdent Pen and QrayCam Pro quantitative fluorescence parameters (ΔFmax and ΔRmax) for occlusal caries assessment in permanent posterior teeth. ICDAS II scores and ICDAS II-derived categories were used as the clinical comparator because histological validation is not feasible in this clinical setting. Therefore, the findings should be interpreted as agreement with clinically derived ICDAS II categories, not as direct evidence of histological lesion depth.

Three main findings emerged. First, DIAGNOdent, ΔFmax and ΔRmax changed progressively across increasing ICDAS II severity. Second, DIAGNOdent and ΔFmax showed higher correlation coefficients with ICDAS II scores and ICDAS II-derived categories than ΔRmax in this sample. Third, ROC analysis showed different descriptive patterns across thresholds: the numerically largest AUCs were observed for ΔFmax when distinguishing sound surfaces from ICDAS II-detected lesions and for DIAGNOdent when separating enamel-category lesions from dentin-suspected lesions. Because formal pairwise AUC comparisons were not performed and the analyses were not adjusted for within-participant clustering, these findings should be interpreted as exploratory descriptive patterns rather than proof of superiority of one device or metric.

The observed patterns are clinically plausible. DIAGNOdent values increased with higher ICDAS II scores, consistent with previous studies reporting higher laser fluorescence readings with increasing visual caries severity on occlusal surfaces [5, 7, 11]. ΔFmax also showed a strong inverse association with ICDAS II severity, reflecting greater fluorescence loss as lesions became more clinically evident. The numerically high AUC for distinguishing sound from affected surfaces suggests that QrayCam-derived fluorescence loss may be useful for documenting departures from sound enamel. However, at more advanced categories, fluorescence-loss measures may show partial saturation, which could help explain why DIAGNOdent showed a numerically larger AUC for the E2-versus-D1 contrast in this sample [19].

ΔRmax showed lower categorical discrimination than DIAGNOdent and ΔFmax, but increased in more advanced ICDAS II categories. This supports interpreting ΔRmax as a complementary red-fluorescence signal rather than as a direct marker of tissue depth. Because red/orange fluorescence is associated with bacterial porphyrins and mature plaque/biofilm, ΔRmax may provide additional information about surface biofilm activity or plaque-retentive sites, especially when interpreted together with ICDAS II and ΔFmax [14–15, 20].

A strength of this study is that calibrated examiners applied ICDAS II across the clinical spectrum studied, and all three device outputs were obtained from the same occlusal sites. ICDAS II is a well-established visual-clinical system with reported reproducibility and correlation with lesion severity in validation studies [3–5]. Nevertheless, it remains a clinical comparator and not a substitute for histology. Compared with previous work, this study adds clinical data for a third-generation QLF camera assessed on the same occlusal sites as DIAGNOdent Pen, including both fluorescence loss and red-fluorescence parameters across ICDAS II codes 0–5 [14–17]. Direct comparison with in vitro studies, including studies using histology as the reference standard, should be made cautiously because of differences in tooth type, study setting, validation method and lesion spectrum [17].

From a practical perspective, the findings do not support using any single numeric output in isolation. ICDAS II should remain the primary clinical framework, while DIAGNOdent and QrayCam Pro may provide adjunctive quantitative information. DIAGNOdent Pen is rapid and chairside-friendly, but readings can be affected by plaque, staining, calculus, hydration, probe angulation and fissure morphology, and the device does not provide spatial mapping of the lesion [6, 11, 21–22]. QLF devices generally provide image-based documentation and spatial information, but requires controlled lighting, standardized positioning and ROI-based analysis, which may introduce operator variability [23]. These different profiles support complementary use rather than a conclusion that one device replaces the other.

### Clinical implications

The clinical relevance of these findings lies in the different potential roles of the three signals. ΔFmax may be useful for documenting fluorescence loss and monitoring early departures from sound enamel. DIAGNOdent may provide additional chairside information when visual findings raise concern about more advanced fissure changes. ΔRmax may help identify sites with prominent biofilm-associated red fluorescence and may support preventive counselling or longitudinal monitoring. However, all three outputs should be interpreted alongside calibrated visual examination, surface conditions, caries-risk assessment and radiographic information when indicated. The exploratory cut-off values reported in this study should not be adopted clinically without external validation.

### Strengths, limitations and future directions

Key strengths were the same-site paired acquisition of DIAGNOdent, ΔFmax and ΔRmax values, the use of calibrated ICDAS II scoring, and the inclusion of a clinically relevant range of occlusal appearances. Several limitations should also be acknowledged. First, the reference standard was ICDAS II-based clinical scoring rather than histology. Second, the tooth was used as the analytical unit, although some participants contributed more than one tooth; because cluster-adjusted models were not fitted, confidence intervals and p-values may be overly optimistic. Third, DIAGNOdent measurements were obtained after ICDAS II scoring and were not fully blinded. Fourth, ICDAS II categories were unevenly distributed, with relatively few code 5 lesions. Finally, this was a single-centre study in a relatively young adult population, which may limit generalisability to other age groups, clinical settings and caries-risk profiles.

Future studies should use prospectively planned sample-size strategies tailored to diagnostic accuracy endpoints, account for within-participant clustering, externally validate candidate cut-offs, and assess whether baseline DIAGNOdent, ΔFmax and ΔRmax values predict lesion progression, stability or arrest over time.

## Conclusions

Overall, DIAGNOdent Pen, ΔFmax and ΔRmax showed descriptive agreement with ICDAS II-derived categories in this exploratory tooth-level clinical study. The three outputs showed different descriptive patterns across predefined contrasts and should be interpreted as complementary adjunctive measures rather than interchangeable diagnostic tests or replacements for calibrated ICDAS II examination. Because analyses were not adjusted for within-participant clustering and formal pairwise AUC comparisons were not performed, the reported confidence intervals, p-values and observed AUC differences should be interpreted cautiously. Numerical cut-offs require external validation before clinical adoption.

## Supplementary Information

Supplementary Material 1.

## Data Availability

The datasets generated and analysed during the current study are available from the corresponding author on reasonable request.

## Abbreviations

AUC: Area under the receiver operating characteristic curve
CI: Confidence interval
ICDAS II: International Caries Detection and Assessment System
QLF: Quantitative light–induced fluorescence
ΔFmax: Maximum percentage fluorescence loss
ΔRmax: Maximum red fluorescence gain (%)
