# Supplementary material for: Clinical cross-sectional diagnostic accuracy study of DIAGNOdent Pen and QrayCam Pro quantitative fluorescence for occlusal caries detection and ICDAS II-derived lesion categorization in permanent posterior teeth
Source: BMC Oral Health. 2026 Jul 4;26:1366. doi: 10.1186/s12903-026-09180-y (PMC13429013; doi:10.1186/s12903-026-09180-y)
Supplement: Supplementary file 1 — Supplementary Material 1. [file 12903_2026_9180_MOESM1_ESM.docx]

Supplementary Table S1. DIAGNOdent Pen values and QrayCam Pro ΔFmax and ΔRmax across ICDAS II codes

| **ICDAS II** | **n** | **DIAGNOdent Pen, median (min–max)** | **ΔFmax (%), median (min–max)** | **ΔRmax (%), median (min–max)** |
| --- | --- | --- | --- | --- |
| **0** | 30 | 10.5 (2–16) | 0.0 (−27 to 0) | 0.0 (0–0) |
| **1** | 39 | 14.0 (6–25) | −17.0 (−53 to 0) | 0.0 (0–48) |
| **2** | 12 | 23.0 (20–40) | −34.0 (−65 to −14) | 0.0 (0–56) |
| **3** | 36 | 45.5 (18–99) | −38.5 (−65 to −9) | 41.5 (0–246) |
| **4** | 23 | 68.0 (30–99) | −60.0 (−77 to −9) | 68.0 (0–149) |
| **5** | 5 | 99.0 (67–99) | −65.0 (−78 to −13) | 281.0 (0–297) |
| **Total** | 145 | 21.0 (2–99) | −27.0 (−78 to 0) | 0.0 (0–297) |

*ΔFmax = maximum percentage fluorescence loss within the lesion area (negative values indicate loss relative to surrounding sound enamel); ΔRmax = maximum percentage increase in the red/green fluorescence ratio within the lesion area relative to surrounding sound enamel.*

Supplementary Table S2. Exploratory ROC-derived cut-off values (maximum Youden index) for detecting any lesion (E0 vs E1+E2+D1)

| **Metric** | **Cut-off rule** | **Sensitivity** | **Specificity** |
| --- | --- | --- | --- |
| **DIAGNOdent Pen** | ≥ 14.5 | 0.809 | 0.967 |
| **ΔFmax (%)** | ≤ −6.5 | 0.983 | 0.900 |
| **ΔRmax (%)** | ≥ 11.5 | 0.487 | 1.000 |

ΔFmax values are negative; more negative values indicate greater fluorescence loss. Therefore, a positive test was defined as ΔFmax ≤ −6.5%. Cut-off values are exploratory and require external validation before clinical use.

Supplementary Table S3. Quantitative values shown in the representative QrayCam Pro examples in Figure 3

| **Representative ICDAS category** | **ΔF (%)** | **ΔFmax (%)** | **ΔR (%)** | **ΔRmax (%)** |
| --- | --- | --- | --- | --- |
| **ICDAS 1** | −6.5 | −9.8 | 21 | 0 |
| **ICDAS 2** | −11.6 | −36.1 | 32 | 64 |
| **ICDAS 3** | −20.9 | −71.2 | 38 | 164 |
| **ICDAS 4** | −30.9 | −76.6 | 74 | 319 |

*Values were transcribed from the QrayCam Pro output panels of the representative examples shown in Figure 3. Decimal commas in the software output were converted to decimal points for consistency with the manuscript. These illustrative values are not additional analytical data and should not be interpreted as diagnostic cut-offs.*
